# Menstrual blood-derived mesenchymal stromal cell extracellular vesicles – a potential tool for tissue regeneration and disease detection

**DOI:** 10.3389/fbioe.2025.1643408

**Published:** 2025-08-08

**Authors:** Raminta Vaiciuleviciute, Jolita Pachaleva, Eiva Bernotiene, Gabija Kugaudaite, Ignas Lebedis, Edvinas Krugly, Ilona Uzieliene

**Affiliations:** ^1^ Department of Regenerative Medicine, Innovative Medicine Centre, Vilnius, Lithuania; ^2^ Department of Chemistry and Bioengineering, VilniusTech Faculty of Fundamental Sciences, Vilnius Gediminas Technical University, Vilnius, Lithuania; ^3^ Department of Environmental Technology, Kaunas University of Technology, Kaunas, Lithuania

**Keywords:** menstrual blood mesenchymal stromal cells, extracellular vesicles, MSCs, biomarkers, therapy, diagnostics

## Abstract

Menstrual blood-derived mesenchymal stromal cells (MenSCs) have emerged as a novel source for regenerative medicine, offering a unique alternative to traditional stem cell types, including adipose-derived and bone marrow-derived mesenchymal stromal cells. MenSCs are characterized by their pluripotency, multi-lineage differentiation potential and immunomodulatory properties, which enable them to contribute to the regeneration of various tissues such as skin, uterus, muscle, connective tissues and nerves. Extracellular vesicles (EVs) secreted by MenSCs contain biologically active molecules, including proteins, lipids, and miRNAs, which play a key role in mediating these regenerative effects. Compared to other MSC-derived EVs, MenSC-EVs offer distinct advantages due to their enhanced regenerative capabilities and lower immunogenicity. Moreover, MenSC-EVs are a promising source for disease biomarkers in various diseases, including female reproductive system issues such as infertility. This manuscript reviews the latest findings on MenSCs and their EVs, highlighting their cargo composition, regenerative potential and as a source of biomarkers across multiple tissues, comparing their cargo profiles with EVs derived from other MSC sources.

## 1 Introduction

Over the past few decades, mesenchymal stromal cells (MSCs) have gained significant attention in regenerative medicine. Traditional sources of MSCs, such as adipose tissue and bone marrow, have been studied and applied in various models due to their multipotency, immunomodulatory properties, and secretion of bioactive molecules, establishing their regenerative potential (Lu et al., 2023; Lei et al., 2013; Tran et al., 2011; Baghaei et al., 2017; Maslennikov and Maksym, 2023; Lee et al., 2013; Asadian et al., 2021; Sober et al., 2023; Xie et al., 2009). However, alternative and relatively less studied sources for MSCs can offer unique advantages over conventional MSC sources. Menstrual blood, accessible without invasive procedures, provides an abundant reservoir of menstrual blood MSCs (MenSCs) possessing multipotency and even pluripotency-like features, including multi-lineage differentiation potential and the ability to promote regeneration of different tissues including the skin, uterus, bones, and muscles (Aleahmad et al., 2018; Rahimi et al., 2018; Mou et al., 2013; Sheikholeslami et al., 2021; Akhavan-Tavakoli et al., 2017; Meng et al., 2007). These features make MenSCs a valuable and a potential candidate for cellular therapy.

MSC extracellular vesicles (EVs), nano-sized particles that encapsulate bioactive molecules such as proteins, lipids and RNA have attracted attention from both scientists and clinicians. Among them, MenSC-EVs have been studied the least. These EVs are key mediators of the regenerative and therapeutic effects of MenSCs, facilitating cellular communication and modulating immune responses (Robalo Cordeiro et al., 2024; Chen et al., 2021; de Pedro et al., 2023). Compared to EVs derived from other MSC sources, MenSCs-EVs exhibit enhanced regenerative properties, a lower immunogenic profile and a greater potential for personalized therapeutic applications. It was shown that MenSC-EVs possess potential wound healing properties, including cardiac, neural, liver tissue repair (Dalirfardouei et al., 2019; Lopez-Verrilli et al., 2016; Wang et al., 2017; Chen et al., 2017) and most importantly–hold promise in female reproductive tissue regeneration (Robalo Cordeiro et al., 2024; Marinaro et al., 2018; Zhang et al., 2021b). Furthermore, the cargo within MenSC-EVs is a potential source for disease biomarkers, offering new strategies in diagnostics and treatment for issues in female infertility and more. For instance, MenSC-EVs can be used for evaluation of endometriosis and endometriosis-related infertility compared to healthy donors (Cordeiro et al., 2023; Zhou et al., 2020). Additionally, undefined female infertility biomarkers can be detected and validated by MenSC-EVs (Vaiciuleviciute et al., 2025).

This review explores the characteristics and functions of MenSCs, comparing them to pluripotency-possessing embryonic stem cells and classical MSCs, focusing on their EVs as a novel therapeutic and diagnostic tool in regenerative medicine. By comparing MenSC-EV cargo to those from other MSC sources, we aim to highlight the unique properties of MenSCs in personalized therapy, tissue regeneration, and disease management, with an emphasis on different disease conditions, such as reproductive system, heart, liver and skin degeneration. Through this review, we illustrate the need for continued research to fully understand the potential of MenSC-EVs, aiming for improved clinical outcomes in the future.

## 2 Menstrual blood-derived mesenchymal stromal cells and their pluripotent-like properties

Endometrial cells exhibiting stemness were first discovered in 2004 (Gargett, 2004) and further characterized as a menstrual-blood stromal cell population in 2007 (firstly referred to as endometrial regenerative cells). MenSCs are collected from menstrual blood, which contains cellular material shed from the functionalis layer of the endometrium during the menstrual phase. This includes endometrial stromal cells and progenitor-like populations with mesenchymal and pluripotency-like features. Unlike amniotic fluid-derived MSCs, which have been shown to originate, at least in part, from exfoliated fetal kidney cells during nephrogenesis and deposited via fetal urine (Rahman et al., 2018), MenSCs represent an adult-derived MSC source from hormonally regulated, cyclic endometrial tissue of two major zones: the functional layer as well as a supportive stroma (Achmad and Götte, 2014).

It was shown that MenSCs possess more advantageous properties compared to BMMSCs, as they are easy to harvest, differentiate into a variety of tissue cells, have a high proliferative rate (doubling every 19.4 h, compared to around 40–45 h for BMMSCs) (Meng et al., 2007) and low immunogenicity (Chen et al., 2019; Gargett et al., 2016; Tabatabaei and Ai, 2017; Liu et al., 2018; Alcayaga-Miranda et al., 2015).

Furthermore, a great advantage of MenSCs is the ability to collect them repeatedly throughout the lifetime during menstruation, presenting potential use for autologous transplantation, and lack of ethical concerns compared to sourcing other types of stem cells. Menstrual blood can be kept at 4°C for up to 3 days with no changes in MenSC morphology, marker expression, proliferation capacity or differentiation potential, adding to the convenience of sourcing them from donors and transporting them prior to isolation and expansion (Liu et al., 2018). Also, an important part of MenSCs is their secretome, which has gained interest as a potential cell-free therapy, while retaining the immunomodulatory, stimulatory and paracrine effects of the cells themselves (Uzieliene et al., 2018; Uzieliene et al., 2023).

Over the last few decades, therapeutic potential of MenSCs has been considered in multiple *in vitro* studies, such as neural, cardiac, liver, lung, endometrium and cartilage diseases (Mou et al., 2013; Toyoda et al., 2007; Azedi et al., 2017; Uzieliene et al., 2023; Skliutė et al., 2021; Valatkaitė et al., 2021). *In vivo* studies also revealed positive results of MenSCs transplantation in the reproductive system. MenSCs transplanted to mice uterus, after endometrial-factor induced infertility, presented a positive impact on endometrium restoration and outcomes (Bausyte et al., 2023). Additionally, it was shown MenSCs increased fertility, number of offspring and restored the estrous cycle of mice after chemotherapy that resulted in ovarian degeneration, indicating the restoration of fertility and ovarian function (Lai et al., 2015). Likewise, a clinical trial with 36 poor ovarian responder women of mature age (>40) was carried out in 2018–2019, implanting autologous MenSCs into the ovaries. The therapy improved oocyte numbers and quality, fertility and overall success of pregnancy (Zafardoust et al., 2020), showing consistent results from MenSC therapy in ovarian health and fertility improvement even in human trials.

### 2.1 Phenotypic profile and differentiation capacity of MenSCs

MenSCs possess a typical MSC phenotypic profile (surface marker expression) compared to other MSCs, although they also express unique, pluripotency-related surface markers. The phenotypic analysis of *in vitro* expanded MenSCs revealed a positive expression for the surface markers CD44, CD73, CD90, and CD105 and negative for CD14, CD34, CD45, CD80, and HLA-DR, while endometrial MSCs have positive expression for CD73, CD90, CD105, CD13, CD29, CD44 markers and the absence of expression of the hematopoietic cell surface antigens CD19, CD34, CD45, CD117, CD130 and HLA-DR (class II) (Zemelko et al., 2012). Moreover, MenSCs possess pluripotency markers, such as Oct-4, SOX2, NANOG, and SALL-4, which make them a unique, MSC type, as compared to other sources MSCs (Borlongan et al., 2010). However, the expression of some pluripotency-associated markers in MenSCs does not equate to the full functional capacity of embryonic stem cells or induced pluripotent stem cells (iPSCs). To date, no definitive evidence has demonstrated the ability of MenSCs to differentiate into all three germ layers *in vivo*, which is a critical hallmark of true pluripotency. Thus, more comprehensive studies, including comparative transcriptomic and functional analyses are needed to validate MenSCs pluripotency, while currently MenSCs remain classified as multipotent.

**FIGURE 1:**
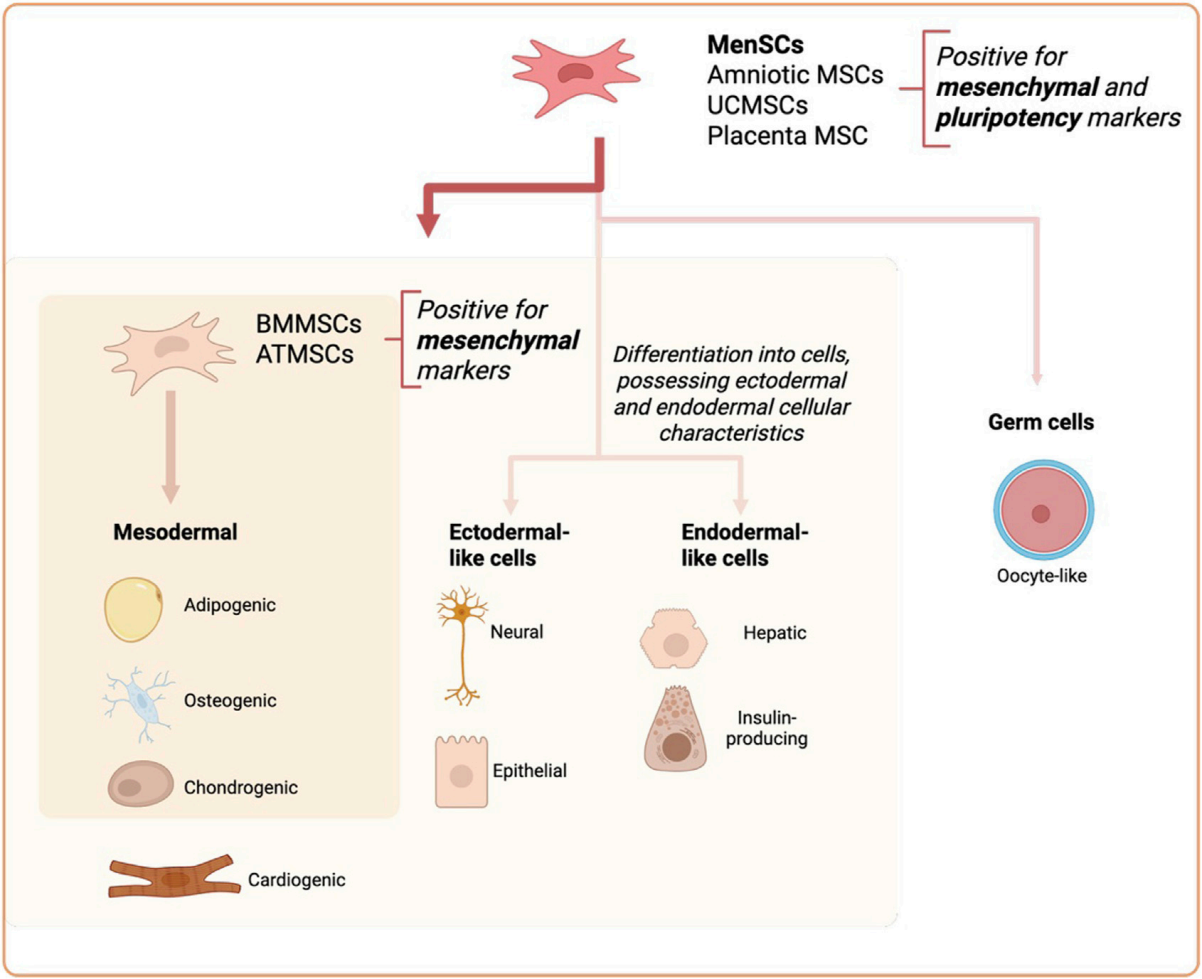
Comparison of MenSCs with Amniotic MSCs, umbilical cord MSCs (UCMSCs), Placenta MSCs, Adipose tissue MSCs (ATMSC) and Bone Marrow MSCs (BMMSC) differentiation potential.

A MenSC surface marker panel, including positive and negative markers (expressed and non-expressed) as well as differentiation capabilities, is provided in Table 1 and summarized in Figure 1, comparing them to embryonic stem cells, BMMSCs, umbilical cord (UC)/Wharton’s jelly, adipose tissue (ATMSCs), amniotic fluid and placental MSCs.

**TABLE 1:** MenSC surface marker expression and differentiation potential, as compared to embryonic, BMMSC, ATMSC, UC/Wharton jelly MSC, amniotic fluid and placental MSCs.

Cell type	Positive markers	Negative markers	Differentiation potential	References
Embryonic stem cells	Pluripotency markers: SSEA-3, SSEA-4, TRA-1-60, TRA-1-81, GCTM2, GCT343, CD9, SOX-2, OCT-4, NANOG, TDGF, GABRB3, DNMT3B,GDF3 class I HLA	Mesenchymal markers CD44	All three embryonic germ layers: mesodermal, ectodermal, endodermal	Nagano et al. (2008) Quintanilla et al. (2014), Reubinoff et al. (2000), Asprer and Lakshmipathy (2015)
MenSCs	Mesenchymal markers: CD9, CD10, CD29, CD44, CD72, CD73, CD90, CD105, and CD146 Pluripotency markers Oct-4, SOX2, NANOG, and SALL-4	Haematopoietic markers CD34, CD38, CD45, CD117, CD133 Endothelial marker: CD31 Antigen: HLA-DR Embryonic marker: SSEA-4 Immune cell marker: CD14, CD80 Cancer marker: CD117 Mesenchymal marker: STRO-1	Chondrogenic Adipogenic Osteogenic Cardiogenic Hepatocyte-like cells Glucose-sensitive beta like-cells Oocyte-like cells Keratinocytes Nucleus pulposus-like cells Myogenic-like Endothelial-like Respiratory endothelial-like Neural-like	Izanlou et al. (2023), Hojjat et al. (2023), Hu et al. (2014), Aleahmad et al. (2018), Rahimi et al. (2018), Mou et al. (2013), Sheikholeslami et al. (2021), Akhavan-Tavakoli et al. (2017), Meng et al. (2007)
BMMSCs	Mesenchymal markers: CD13, CD29, CD44, CD58, CD71, CD73, CD90, CD105, CD106 CD146, CD166, CD271	Haematopoietic markers: CD34, CD45 Immune cell marker: CD14, CD19 Endothelial marker: CD31 Antigen: HLA-DR	Chondrogenic Adipogenic (brown fat) Osteogenic	Lu et al. (2023) Re et al. (2023)
UC/Whartton’s jelly-MSC	Mesenchymal markers CD29, CD44, CD51, CD56, CD73, CD90 CD105, CD146, CD166 Pluripotency markers: low levels of Tra-1-60, Tra-1-81, NANOG, OCT-4, SSEA-3, SSEA-4	Immune cell marker: CD14, CD19 Haematopoietic markers: CD34, CD45 Antigen: HLA-DR	Chondrogenic Adipogenic Osteogenic Epithelial-like Neural-like Hepatic-like Myogenic-like Cardiac-like Insulin-producing cells Oocyte-like cells	Wang et al. (2004), Margossian et al. (2012), Maslennikov and Maksym (2023), Sober et al. (2023), Toyota et al. (2021), Abouelnaga et al. (2022), Majore et al. (2011), Chen et al. (2016)
ATMSCs	Mesenchymal markers: CD9, CD10, CD13, CD73, CD29, CD49e, CD54, CD55, CD79a CD166 and ALCAM, CD44, CD144, CD90, CD105, CD146, CD106, (HLA)-ABC, CD271	Haematopoietic markers: CD45 Endothelial marker: CD31 Immune cell marker: CD14, CD11b, CD19, CD56 Melanoma marker: CD146	Adipogenic (brown fat) Osteogenic Chondrogenic	Yang et al. (2024), Rebelatto et al. (2008)
Amniotic fluid MSCs	Mesenchymal markers: CD29, CD 90, CD105, CD73, CD44, CD166 Pluripotency markers: Oct-4, SSEA-4, c-Myc	Immune cell marker: CD14 Endothelial marker: CD31 Haematopoietic markers: CD34, CD45, CD117	Chondrogenic Osteogenic Adipogenic Myogenic-like Neural-like Oocyte-like cells Keratinocytes Insulin-producing Hepatic-like Vascular endothelial-like	Shamsnajafabadi and Soheili (2022), Portmann-Lanz et al. (2006), Sober et al. (2023), Maslennikov and Maksym (2023), Toyota et al. (2021), Abouelnaga et al. (2022), Yu et al. (2014); Lan et al. (2020), Zheng et al. (2008), Markmee et al. (2017), Mu et al. (2017), Naeem et al. (2022)
Placental MSCs	Mesenchymal markers: CD105, CD146, CD29, CD73, CD90, MHC I, CD49a, CD105, CD106, CD13, CD166, CD146, HLA-ABC Pluripotent markers: SSEA-4, mRNA of Nanog, Sox2, Rex-1	Immune cell marker: CD14, CD40, CD80 and CD86 Haematopoietic markers: CD34, CD45 Endothelial marker: CD31 Antigen: HLA-DR BMMSC marker: CD271	Chondrogenic Osteogenic Adipogenic Myogenic-like Neural-like Cardiac-like Hepatic-like Insulin-producing Oocyte-like	Vellasamy (2012), Castrechini et al. (2010), Tran et al. (2011), Abumaree et al. (2013), Maslennikov and Maksym (2023), Sober et al. (2023), Portmann-Lanz et al. (2006), Toyota et al. (2021), Abouelnaga et al. (2022), Roberts et al. (2019), Chien et al. (2006), Sun and Ji (2009), Asgari et al. (2017)

Beside phenotypical differences with other types of stem cells, MenSCs also differ in their differentiation capabilities. It is known that MenSCs differentiate into a wide range of cell types, and are even able to differentiate into cardiomyocytes with the functions of spontaneously beating cells after induction, resulting in the decreased myocardial infarction area in a rat model (Hida et al., 2008; Ikegami et al., 2010). Furthermore, it has been shown that MenSCs are capable of differentiation into neural, epidermal-like cells (Azedi et al., 2017; Faramarzi et al., 2016; Chen et al., 2015; Toyoda et al., 2007), functional hepatocytes (Mou et al., 2013) and even oocyte-like cells (Asgari et al., 2017) which suggest a superior spectrum of their differentiation potential compared to other tissue MSCs.

### 2.2 MenSCs secretome

MenSCs secrete large amounts of paracrine factors, including growth factors responsible for endometrium regeneration, which may be a potential co-stimulant for other tissue regeneration purposes (Chen et al., 2019; Liu et al., 2018). MenSCs also secrete angiogenic factors VEGF, HGF, ANG and MMP-1 and different cytokines (IL-6, IL-8 and IFN-gamma), and most importantly, MenSCs were shown to be safe to transplant due to their low tumorigenicity (Liu et al., 2018). The secretome of MenSCs also includes EVs, containing proteins or miRNAs (more information in section 2). Different studies reported secretion of various growth factors by MenSCs. Table 2 summarizes all current findings on MenSCs secretome, including protein family, functions and comparison to other types of MSCs.

**TABLE 2:** MenSCs secreted proteins and comparison to other types of MSCs.

Family	Factors	Function	MenSC secretome comparison to other MSC types	References
Epidermal Growth Factor (EGF) Family	EGF	Stimulate cell growth, proliferation, and differentiation	Not compared	Jiang and Wang, (2012)
Fibroblast/keratinocyte Growth Factor (FGF/KGF) Family	bFGF KGF		MenSCs secrete more than BMMSCs	Alcayaga-Miranda et al. (2015), Ren et al. (2016)
Vascular Endothelial Growth Factor (VEGF) Family	VEGF, angiopoietin-1 (Ang-1), angiopoietin-2 (Ang-2), Stromal-derived factor-1 (SDF-1)	vascular remodeling and angiogenesis	No difference in secretion of Ang-1, BMMSC secrete more Ang-2 compared to MenSC No differences in secretion of SDF-1, VEGF between MenSC and BMMSC MenSCs had higher VEGF secretion than dental pulp MSCs in early passages	Manshori et al. (2022), Jiang and Wang (2012), Li et al. (2023), Li et al. (2019)
Transforming Growth Factor-Beta (TGF-β) Family	TGF-β, Growth differentiation factor 15 (GDF-15)	Cell growth, differentiation, and apoptosis	Not compared	Jiang and Wang (2012), Li et al. (2023)
Neurotrophin (NT) Family	Brain-Derived Neurotrophic Factor (BDNF), β-Nerve Growth Factor (β-NGF), Neurotrophin-3 (NT-3), Neurotrophin-4/5 (NT-4/5), Artemin (ARTN), Glial cell line-derived neurotrophic factor (GDNF), Neurturin (NTN), Prospero Homeobox Protein 1 (PSPN), Cerebral Dopamine Neurotrophic Factor (CDNF), Mesencephalic Astrocyte-Derived Neurotrophic Factor (MANF)	Promote survival and differentiation of neurons	Not compared	Li et al. (2019)
Hepatocyte Growth Factor (HGF) Family	HGF	Cell growth, cell motility, and morphogenesis	No differences in secretion of HGF between MenSC and BMMSC; MenSCs had a higher secretion than dental pulp MSCs	Manshori et al. (2022), Li et al. (2019), Ren et al. (2016)
Insulin-like Growth Factor (IGF) Family	IGF-1	Particularly muscle and bone growth and development	Not compared	Li et al. (2019)
Inhibitors of Apoptosis (IAP) Family	X-linked Inhibitor of Apoptosis Protein (XIAP)	Suppresses apoptosis	Not compared	Li et al. (2023)
Hypoxia-Inducible Factor (HIF) Family	Hypoxia-Inducible Factor 1 α (HIF-1α)	Cellular response to low oxygen conditions, involved in processes like angiogenesis and metabolism	MenSC secrete more than BMMSC	Manshori et al. (2022)
Thrombospondins Family	Thrombospondin-1, -2, and -5	Play roles in angiogenesis, tissue remodeling, and cell adhesion	Not compared	Li et al. (2023)
Interleukins (IL)	Interleukin-1β (IL-1β)	Involved in the inflammatory response	BMMSC secrete more than MenSC	Manshori et al. (2022)
Colony-stimulating Factor (CSF)	Granulocyte-macrophage colony-stimulating factor (GM-CSF)	Immune and inflammatory response, MSC mobilization and migration	MenSC secretion significantly higher than umbilical cord MSC	Meng et al. (2007), Kim et al. (2019)

Factors secreted by MenSCs already showed positive immunomodulatory, cardioprotective, angiogenic and regenerative effects. Paracrine effects of MenSCs were analysed in numerous studies and their effects were proposed being more superior to BMMSCs (Alcayaga-Miranda et al., 2015). For instance, MenSC paracrine factors possessed promising results in rat model of myocardial infarction by reducing apoptosis of cells and stimulating endogenous regeneration, while transplantation of MenSCs achieved significantly better cardiac performance than BMMSCs or ATMSCs (Jiang et al., 2013; Wang et al., 2017). Important to note, BMMSCs were shown to secrete higher concentrations of IL-1β (Jiang and Wang, 2012; Manshori et al., 2022). Also, it was revealed that MenSC secrete higher amounts of EGF, FGF and HIF-1α, as compared to BMMCS, while no differences were observed in VEGF or angiopoietin secretion, which were higher in the MenSC secretome compared to UC and dental pulp MSCs. Moreover, MenSC secrete higher levels of HGF than dental pulp MSCs and higher levels of GM-CSF compared to UCMSCs.

Noteworthy, MenSC secretome can be modulated by different environmental conditions. For instance, under hypoxic conditions MenSCs secreted significantly higher levels of VEGF, while EGF and TGF-β secretion was not affected (Jiang and Wang, 2012; Alcayaga-Miranda et al., 2015). Hypoxia can also enhance the release of EVs, as previously shown in UCMSCs (Zhang et al., 2012). Moreover, it was demonstrated that endometrial MSC MiRNAs: miR-148a-3p, hsa-miR-378a-3p (related to angiogenesis, wound healing), hsa-miR-424-5p (associated with angiogenesis), hsa-miR-23a-3p, and hsa-miR-let-7a-5p (related to immune modulation) were the most widely expressed in acute hypoxic conditions (0.1%-1%), while hsa-miR-34a-5p (reduces expression of VEGF), hsa-miR-532-5p, hsa-miR-221-3p, hsa-miR-93-5p (regulating cell cycle and proliferation) were detected only under normoxic conditions (de Pedro et al., 2023). These results are directly associated with MenSC physiological behavior *in vivo* and differences obtained *in vitro*.

In order to stimulate MenSC immunomodulator or regenerative properties, MenSCs can be additionally stimulated by external factors using different cultivation conditions. MenSCs increase IDO1 secretion and EVs release under treatment with IFN-γ and TNF-α (de Pedro et al., 2021). bFGF and 5-aza increased the levels of VEGF, SDF-1, HIF-1α, IL-1β, and ANG-1 secretion from MenSCs (Manshori et al., 2022). Moreover, MenSCs may help protect insulin-producing pancreatic β-cells from autoimmune attack in type 1 diabetic mice. By modulating immune responses, these cells could potentially slow disease progression and preserve insulin production (Wu et al., 2014).

## 3 MenSC EVs and their cargo

**FIGURE 2:**
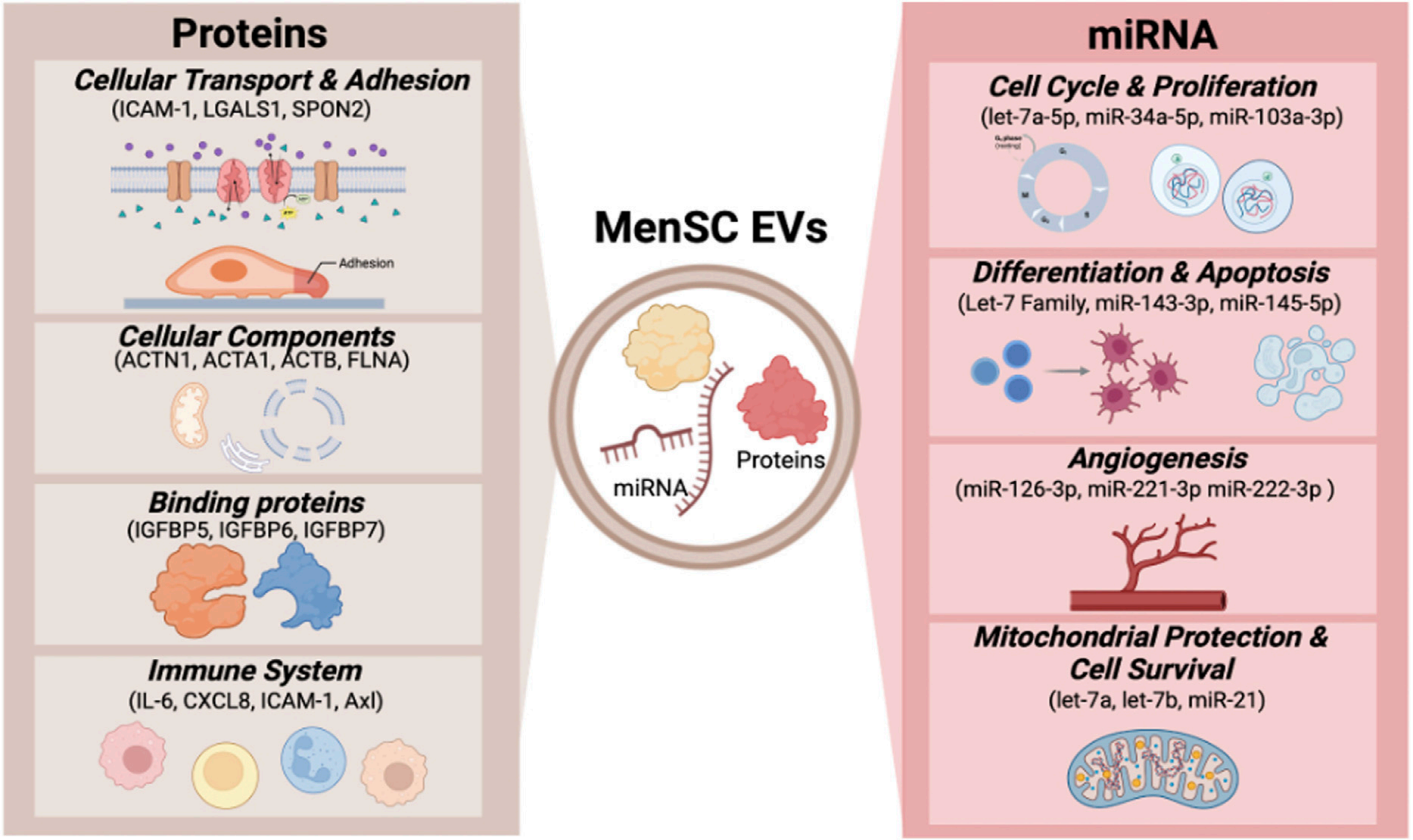
MenSC-EV cargo.

EVs isolated from human bodily fluids, such as blood, urine, saliva, or cell culture supernatants have emerged as a promising approach for non-invasive therapies and diagnostics, also known as “liquid biopsy” because of their selectively packed cargo, including proteins, lipids and nucleic acids (Yokoi et al., 2015; Jia et al., 2014; Ciferri et al., 2021; Matsuzaka and Yashiro, 2022). The cargo of EVs is essential for cellular responses and can regulate various physiological and pathological processes, as well as serve as potential biomarkers for diagnosis (Mir and Goettsch, 2020). MenSC-EVs have demonstrated regenerative properties, primarily due to their capacity to transport cargo to recipient cells and modulate key signaling pathways associated with cell survival, differentiation, and proliferation (Robalo Cordeiro et al., 2024; Chen et al., 2021; de Pedro et al., 2023). A schematic representation of MenSC-EV composition is presented in Figure 2.

The composition of EV cargo is highly specific and depends on the cell type, metabolic state, and presence of disease. Furthermore, the cargo of EVs is the main factor that defines their mechanism of action, application possibilities, and therapeutic effects (Mir and Goettsch, 2020; Figueroa-Valdés et al., 2021).

### 3.1 MenSCs and other MSC EV protein cargo

Mainly, MenSC-EVs carry proteins related to processes such as cellular transport, including vesicle-mediated transport or cell adhesion and migration. An additional group of proteins is related to cellular components, including extracellular organelles, membrane components and parts of the cytosol. MenSC-EVs are also enriched with different binding proteins. Upon evaluation of the functional properties of the most abundant proteins in MenSC-EVs, it was determined that the majority are associated with immune system processes and extracellular matrix (ECM) organization (de Pedro et al., 2023). Additionally, MenSC-EVs contain various bioactive molecules, including cytokines. A comparative analysis of MenSCs and MenSC-EVs revealed that the latter contain higher concentrations of IL-6 and IL-8, intercellular cell adhesion molecule-1 (ICAM-1), angiopoietin-2, Axl, angiogenin, insulin-like growth factor-binding protein 6 (IGFBP-6), and osteoprotegerin (Chen et al., 2017). Moreover, it was reported that MenSC-EV are enriched with E3 ubiquitin ligase (UBR4), which inhibited fibrosis of rat endometrial stromal cells by affecting YAP activity (Qi et al., 2023).

The culturing conditions of MenSCs significantly alter the cargo and the EV-associated proteome. Proinflammatory conditions were found to downregulate proteins related to wound healing, adhesion and migration processes and upregulate proteins involved in angiogenesis and inflammatory responses. As an example, MenSCs cultured under physioxic conditions (1%–2% O_2_) secreted EVs enriched with proteins related to cell adhesion and intracellular transport. Acute hypoxia (<1% O_2_) had different effects on EV cargo–it upregulated proteins associated with cell adhesion, cell migration and angiogenesis pathways (de Pedro et al., 2023).

At present, the available information regarding MenSC-EV cargo is relatively limited in relation to MSC-derived EVs from alternative sources, such as ATMSCs, BMMSCs, and UCMSCs. BMMSC-EVs contain proteins involved in ion and other protein transport. In addition, proteins associated with cell cycle regulation, transcription and translation regulation, cell adhesion and lipid metabolism, apoptosis and inflammation were identified in BMMSCs. Upon classification of proteins according to cellular components, the majority of proteins were found to be associated with the cell membrane, nucleus, cytoplasm, mitochondria and endoplasmic reticulum (McBride et al., 2021).

ATMSC-EVs encompass a multitude of proteins which play crucial roles in various biological processes. These processes include cellular migration, modulation of immune responses, proliferation of cells, formation of new blood vessels, metabolism of osteocytes, and regeneration of nerve tissue (Alonso-Alonso et al., 2022).

Human UCMSC exosomes are enriched with proteins related to different mechanisms and signaling pathways. The majority of proteins detected in UCMSC exosomes play roles in modulating various biological processes, including complement response, HIF-1, MAPK signaling, metabolic pathways, NF-κB pathway, and microbial infection. Additionally, proteins related to PI3K-AKT, cholesterol metabolism, IgA production, VEGF, and B-cell receptor signaling pathways were detected (Bi et al., 2022). Table 3 presents a more detailed categorization of cargo proteins in different MSC-derived EVs and their respective functions.

**TABLE 3:** Comparison of EVs protein cargo from different sources of MSCs.

Source of EVs	Method of EV isolation	Proteins	Function	References
MenSCs	Ultracentrifugation (UC)	N/A	Protein, ATP, RNA, enzyme, collagen, cadherin binding ECM organisation ECM-receptor interaction Platelet activation, signaling and aggregation Elastic fibre formation Non-integrin membrane-ECM interactions GPER1 signaling Post-translational protein phosphorylation B cell receptor signaling pathway Positive regulation of cell motility L1CAM interactions Acute inflammatory response Basement membrane organisation Complement system Response to hormone/steroid hormone Humoral immune response ADP metabolic process	de Pedro et al. (2023)
	ExoQuick-TC	IL-6, IL-8, ICAM-1, Axl, IGFBP-6	Inflammatory and Immune Response	Chen et al. (2017)
	Filtration/centrifugation combination	COL1A1, COL1A2, COL3A1, COL5A1, COL5A2, COL6A1, COL6A3, COL12A1, LUM, ECM1, SPARC, TGFBI, PCOLCE	ECM and Structural Proteins	Marinaro et al. (2019)
		VIM, ACTN1, ACTN4, ACTA1, ACTB, FLNA, VCL, MYH9, TPM1, TPM4, PFN1	Cytoskeletal and Structural Proteins	
		LGALS1, LGALS3BP, ISLR, SPON2, CLSTN1	Cell Adhesion and Signaling Proteins	
		TIMP1, TIMP2, MMP2, MMP3, SERPINF1, CST3, A2M	Protease Inhibitors and Enzymatic Regulators	
		ALB, HPX, PSAP, NUCB1, PPIA, PPIB	Plasma and Transport Proteins	
		IGFBP5, IGFBP7, DKK3	Growth Factor Binding Proteins	
		THBS1, C1S, C1R, NID1, PTGDS, TAGLN	Functional Proteins	
BMMSCs	ExoQuick-TC^®^ ULTRA EV Isolation Kit UC	CACNA1G, CACNA1H, CACNB2, RYR1, ATP2C1, S100A8	Calcium transport-related proteins	McBride et al. (2021) Pomatto et al. (2021)
	UC	SCN4A, SCN10A, TRPM2	Sodium-related channels	Pomatto et al. (2021)
		RPB1, MINA	Transcriptional regulators	
		CCL2, CSF3, CXCL1, CXCL9, IL-10	Inflammatory and Immune Response	
		FN1, THBS1	Extracellular matrix and cell adhesion	
		EPO, PDGFRA, NRG3, RET	Growth factors	
		LYN, TEC	Signal Transduction and Kinases	
		ALDOA, MAN2B1	Metabolism and Enzymes	
		APOA4, NPTX1, POMC, MUSK	Neural and Neuroendocrine Function	
ATMSCs	UC	ADGRB1, IL1R1, IL1RL1, IL2RB, LHCGR, PDGFRB, TNFRSF13C, TNFRSF8	Receptors and Signal Transduction Receptors involved in cell communication, proliferation, immune response and immune cell activation	Pomatto et al. (2021) Xing et al. (2020)
		ANGPT1, BMP5, BMP7, FGF10, FGF16, FGF18, GDF1	Growth Factors and Developmental Proteins	
		C2, CCHCR1, CCL19, CCL28, CCL4, CSF2RA, CXCL2, LAG3, LTA, TNF	Immune system proteins	
		CLU, DKK4, MMP20, MUC16	Extracellular Matrix and Structural Proteins	
		ALPP, CHI3L1, CKB, EPX, HRG, IAPP	Metabolic and Enzymatic Proteins	
		UBB	Ubiquitin-Related Protein	
UCMSCs	UC	TALDO1, LDHA, ENO1	Metabolism and Energy Production	Bi et al. (2022)
		MARCKS, DSTN, CFL1, MSN, CDC42, NRAS	Cytoskeletal and Structural Proteins	
		YWHAZ, YWHAH, YWHAG, NAP1L1, EIF4A1, SRI, PRDX6	Signaling and Regulatory Proteins	
		SLC44A2, SLC39A14, SLC1A5, RAB11B, TSPAN4	Membrane and Transport Proteins	
		HSPAB, CNDP2, PPIA	Stress Response and Enzymatic Proteins	
	UC	FN1, EMLIN1, OLFML3, ITGA4	ECM and Adhesion Proteins	Bi et al. (2022)
		CORO1A, DNMIL, FARP1	Cytoskeletal and Structural Proteins	
		APOE, APOC3, PLTP, PYGB	Plasma and Transport Proteins	
		PRKAR28, PPPICB, GNAO, RABSA, NAPA	Signaling and Regulatory Proteins	
		JCHAIN, C4B	Immune System and Complement Proteins	
	UC	COL6A1, COL6A2, COL6A3, EDIL3, ITGA6, ITGB1, ITGA2, ITGA2B, ILK, TLN1, FERMT3	ECM and Structural Proteins	Bi et al. (2022)
		ACTC1, ACTN1, ACTR3, ANXA1, ANXA3, ANXA7, ANXA11, ARPC1B, ARPC2, CAPZA1, CAPZB, CNN2, FLNA, GAPDH, PGK1, PFKP, RAP1B, ROCK2, SRC, TPM4, VCL, WASF2, WDR1	Cytoskeletal and Structural Proteins	
		CD9, CLU, CORO1C, FCGBP, LGALS1, LGALS3BP, SND1, STXBP2, TAGLN2	Cell Adhesion and Signaling Proteins	
		A2M, ADAM10, SERPIND1, SERPINE1, SERPINE2, ITIH2, ITIH4, MME, C3, C1R, F13A1	Protease Inhibitors and Enzymatic Regulators	
		A2M, APOA1, APOA4, APOB, APOC1, APOD, APOL1, CEMP, CP, FGA, F5, HP, Gc, HBA1	Plasma and Transport Proteins	
		ADH5, CBR1, GAPDH, PFKP, PKM, PGK1	Metabolism and Energy Production Proteins	
		ARF4, RAB1A, RAB7A, RAB14	GTPases and Vesicular Transport Proteins	
		HSP90B1, STOM	Heat Shock and Stress Response Proteins	
		AP1B1, CAP1, CLTC	Clathrin and Vesicle Trafficking Proteins	
		HLA-A, IGHM	Immune System and Complement Proteins	

### 3.2 MenSCs and other MSC EV miRNA cargo

MenSC-derived EVs contain a broad range of microRNAs (miRNAs). The identified mi RNAs in MenSC-EVs included let-7a-5p, miR-143-3p, miR-21-5p, let-7b-5p, let-7f-5p, miR-16-5p, miR-199a-3p, miR-199b-3p, miR-126-3p, let-7i-5p, miR-26a-5p, which are involved in the regulation of cell cycle, proliferation, differentiation, apoptosis and angiogenesis (Marinaro et al., 2019). Let-7 and miR-21 play crucial roles in controlling mitochondrial-DNA damage, promoting cell survival and proposed to provide superior cardioprotection and alleviate pulmonary fibrosis (Wang et al., 2017; Sun et al., 2019). Cargo of MenSC-EVs also contain information related to certain diseases and their predispositions to them. The elevated levels of miR-4443 found in MenSC-EVs were discovered to play a role in the progression of endometriosis. This specific miRNA was found to suppress ACSS2 expression and as a result activate the PI3K/AKT signaling pathway. This activation resulted in enhanced migration and proliferation of endometrial stem cells (Ji et al., 2024).

An analysis of EVs from MenSCs, BMMSCs, and ATMSCs revealed that MenSC-EVs exhibited the highest levels of miR-21 among the three sources of EVs. Additionally, the paracrine effect of MenSCs on rat myocardial infarction was compared to that of BMMSCs and ATMSCs. MenSCs were found to enhance cardioprotection through the transfer of miR-21 via EVs. miR-21 from menstrual blood EVs downregulated phosphatase and tensin homolog (PTEN), enhancing Akt survival kinase activity, resulting in reduced apoptosis in cardiomyocytes and improved angiogenesis in endothelial cells (Wang et al., 2017). This finding demonstrates the superior cardioprotective effect of MenSC-EV cargo compared to BMMSC or ATMSC EVs. Additionally, EVs derived from MenSCs attenuate severe pulmonary inflammation and damage through the transmission of miRNA-671. This miRNA is known to target the kinase AAK1 for post-transcriptional degradation. AAK1 positively regulates the NF-κB signaling pathway (Lian et al., 2023).

MiRNAs highly expressed in BMMSC-EVs were found to be associated with cellular proliferation, death, metabolism and immune regulation (miRs-21, miR-22, miR-26a, miR-10b, miR-99b, miR-125b, and miR-148a) (Vaka et al., 2023; Baglio et al., 2015). In comparison with BMMSC-EVs, UCMSC-EVs are enriched with miRNA related to regenerative processes, aging and cell proliferation (Vaka et al., 2023). Several studies reported, that the most abundant miRNA in UCMSC-EVs are miR-16, miR-21, miR-23, miR-34, miR-146a and miR-222, which are associated with cell proliferation and immune regulation (Jothimani et al., 2022). MenSC-EV and other MSC EV miRNAs are presented in Table 4.

**TABLE 4:** Comparison of EVs miRNA cargo from different sources of MSCs.

Source of EVs	Method of EV isolation	miRNA	Function	References
MenSCs	MagCapture Exosome isolation kit	let-7	Cell Cycle and Proliferation, apoptosis and tumor suppression	Sun et al. (2019)
	UC	miR-21	Cell Cycle and Proliferation, Apoptosis and Tumor suppression	Wang et al. (2017)
	UC	miR-4443	Inflammation	Ji et al. (2024)
	UC	miR-671	Neuroprotection	Lian et al. (2023)
	Filtration/centrifugation combination	let-7a-5p, let-7b-5p, let-7f-5p, let-7c-5p, let-7i-5p, let-7e-5p, let-7g-5p miR-21-5p, miR-126-3p, miR-126-5p, miR-223-3p, miR-103a-3p, miR-486-5p	Cell Cycle and Proliferation	Marinaro et al. (2019)
		miR-143-3p, miR-145-5p, miR-34a-5p, miR-155-5p, miR-203a	Apoptosis and Tumor Suppression	
		miR-126-3p, miR-126-5p, miR-221-3p, miR-222-3p	Angiogenesis and Vascular Regulation	
		miR-142-3p, miR-142-5p, miR-223-3p, miR-155-5p, miR-451a	Inflammation and Immune Response	
		miR-122-5p, miR-425-5p, miR-191-5p	Metabolism and Homeostasis	
		miR-125b-5p, miR-125a-5p, miR-23a-3p, miR-23b-3p, miR-26a-5p, miR-26b-5p, miR-30a-5p, miR-30d-5p, miR-30e-5p	Stem Cell Regulation and Differentiation	
BMMSCs	UC	let-7a-5p, let-7e-5p, miR-197-3p, miR-342-3p, miR-99a-5p	Tumor Suppression and Cancer Regulation	Pomatto et al. (2021)
		miR-483-5p miR-484	Inflammation and Immune Response	
		miR-130b-3p, miR-199a-3p, miR-365a-3p, miR-365b-3p	Cell Proliferation, Differentiation and Apoptosis	
		miR-10b-5p miR-29b-3p miR-483-5p	Metabolism and Organ Development	
	UC	miR-199a-3p miR-23a-3p let-7b-5p let-7a-5p, miR-125b-5p	Tumor Suppression and Cancer Regulation	Vaka et al. (2023)
		miR-155-5p	Inflammation and Immune Response	
		miR-877-5p miR-4454	Metabolic Regulation and Cellular Homeostasis	
ATMSCs	UC	miR-10a-5p miR-125b-1-3p, miR-126-5p, miR-129-2-3p, miR-136-3p, miR-137, miR-140-5p, miR-144-5p, miR-145-3p miR-148a-3p, miR-148b-5p, miR-149-5p	Tumor Suppression and Cancer Regulation	Pomatto et al. (2021) Xing et al. (2020)
		miR-142-3p, miR-181a-2-3p	Immune System and Inflammation	
		miR-1291	Metabolism and Drug Resistance	
		miR-1226-5p miR-1270	Cell Proliferation, Differentiation and Apoptosis	
UCMSCs	UC	miR-21-5p, miR-423-5p	Oncogenic miRNAs and Cancer Progression	Bi et al. (2022)
		miR-146a-5p	Inflammation and Immune Response	
		miR-320a-3p	Cardiovascular and Metabolic Regulation	
		let-7i-5p	Tumor Suppression and Cell Cycle Regulation	
	UC	miR-125b-5p, and miR-145-5p	Tumor Suppression and Cancer Regulation	Shi et al. (2021)
		miR-23a-3p	Cell Proliferation, Apoptosis and Differentiation	
	UC	miR-125b, miR-199a-3p, miR-765, miR-28-5p, miR-100-5p	Tumor Suppression and Cancer Regulation	Vaka et al. (2023)
		miR-223-3p	Inflammation and Immune Response	
		miR23a-3p, miR-4454	Cellular Homeostasis and Stress Response	

## 4 MenSC-EV potential in tissue regeneration

MenSC-EVs showed promising therapeutic potential for regeneration of different tissues, but the most significant effects were demonstrated for the regeneration of female reproductive system tissues, including endometrium and ovaries in diseases, such as Premature Ovarian Insufficiency (POI) and Intrauterine Adhesion (IUA). In addition to that, numerous studies have also indicated the MenSC-EV therapeutic potential on wound healing, neural, liver, heart tissue repair and more.

### 4.1 MenSCs-EV therapeutic effect on female reproductive tissues

In a rat model of POI induced by chemotherapeutic agents, MenSC-EVs restored ovarian function by increasing ovarian weight, follicle numbers at various developmental stages, and serum estrogen levels. The therapeutic effects were associated with activation of the PI3K/AKT signaling pathway, inhibition of apoptosis, and overall enhancement of ovarian microenvironment stability (Robalo Cordeiro et al., 2024). MenSC-EVs contribute to endometrial repair by enhancing cell proliferation and stimulating VEGF production, which promotes angiogenesis (Marinaro et al., 2018). A study by Zhang et al. demonstrated that MenSC-EVs had the effect of promoting ovarian cell proliferation, inhibiting apoptosis and regulating the ovarian extracellular matrix, while increasing the expression of follicle markers DAZL and FOXL2 in rat ovaries. Also, MenSC exosome injections restored the female rat estrous cycle and increased fertility, as treated subjects exhibited increased endometrial thickness, improved glandular formation and reduced fibrosis. Notably, repeated EV administration enhanced endometrial receptivity and improved embryo implantation rates, suggesting potential clinical applications in infertility treatment (Zhang et al., 2021b).

### 4.2 MenSCs-EV therapeutic effect on other tissues

MenSC-EVs have demonstrated efficacy in wound healing by promoting fibroblast proliferation, collagen synthesis, and reducing oxidative stress (Zhang et al., 2023). MiRNA cargo, including miR-21 and miR-29, facilitates keratinocyte migration and differentiation, accelerating skin repair. In models of skin injury, MenSC-EVs have been shown to accelerate wound by promoting the growth of new skin cells (keratinocytes and fibroblasts), increasing collagen production. MenSC-EV treatment promoted re-epithelialization, increased angiogenesis, and modulated inflammation, leading to improved healing outcomes in a diabetic mouse model (Dalirfardouei et al., 2019). These effects suggest their potential use in treating chronic wounds or burns.

MenSC-EVs have also shown ability to promote axonal regeneration and functional recovery following neural injury, further underscoring their broad therapeutic utility (Lopez-Verrilli et al., 2016). Moreover, MenSC-EVs can alleviate fulminant hepatic failure. In experimental models, these EVs reduced liver inflammation and promoted hepatocyte proliferation, leading to improved liver function (Chen et al., 2017). Additionally, MenSC-EVs have been shown to promote angiogenesis and reduce scarring in heart tissue, ultimately improving heart function and reducing long-term damage (Wang et al., 2017). And as mentioned before, MenSC-EVs may help slow down fibrosis by reducing the activity of fibroblasts (cells that contribute to scarring) and lowering levels of fibrotic markers, leading to improved lung function as well (Chen et al., 2021).

In cancer therapy, it was shown that MenSC-EVs block tumor associated angiogenesis and could be used as a tool for cancer treatment. MenSC-EVs reduced the secretion of VEGF and NF-κB activity in human prostate PC3 tumor cells (Alcayaga-Miranda et al., 2015). Other studies additionally emphasize the pro-angiogenic effect of MenSC-EVs (Chang et al., 2021a; Wang et al., 2017). Therefore, the precise mechanism of this targeted action of MenSC-EVs remains unclear.

MenSC-EVs also exhibit immunomodulatory properties by regulating T-cell proliferation, macrophage polarization, and inflammatory cytokine production (Song et al., 2023; Qi et al., 2023). This suggests potential therapeutic applications in autoimmune diseases, inflammatory disorders, and systemic tissue repair.

## 5 MenSC and menstrual blood EVs–a source for disease biomarkers and future diagnostic strategies

EVs show a great potential in diagnostics of different pathologies with leading studies related to early cancer detection, monitoring tumor progression and response to treatment (Weng J. et al., 2021; Kumar et al., 2024). EVs also showed promising results in detection of neurodegenerative diseases (Parkinson’s disease, Alzheimer disease) as they had increased levels of tau proteins, contributed to the diagnosis of cardiac diseases (cardiac fibrosis, ischemic heart disease, heart failure and others) with increased levels of miR-133a, miR-499, miR-199a, pregnancy disorders with higher numbers of circulating EVs in preeclamptic and eclamptic women (Ciferri et al., 2021; Liu et al., 2021; Zhang et al., 2022; Smith and Russell, 2022). EVs could potentially improve diagnostic accuracy and further treatment decisions. The main advantages of EVs for diagnostic approaches include stability in circulation and ability to protect their cargo (Kodam and Ullah, 2021). Nevertheless, challenges remain in EV isolation, especially from human biofluids. Their characterization needs advanced analysis methods, such as digital PCR, mass spectrometry, also in addition to proper storage to keep them intact for clinical application (Weng Z. et al., 2021; Jia et al., 2014; Kodam and Ullah, 2021). Despite these challenges, EVs hold significant promise for improving disease diagnosis and monitoring.

EVs isolated from various reproductive biofluids, including follicular fluid, uterine fluid, peritoneal fluid and serum, alongside the endometrium and endometrial lesions have exhibited significant alterations in miRNAs in pathological conditions such as PCOS premature ovarian insufficiency, endometriosis, and recurrent spontaneous abortion (Duval et al., 2024; Esfandyari et al., 2021). However, conventional diagnostic procedures, such as endometrial biopsies and follicular fluid collection are often invasive, painful, and associated with potential complications (Terzic et al., 2022), while MenSC or menstrual blood serum EVs could be used for the analysis of uterine lesions and abnormalities.

Beyond the regenerative capabilities of MenSC-EVs, these vesicles also hold promise in disease diagnostics and biomarker discovery. Their cargo can provide valuable insights into the molecular changes associated with aging and disease progression. Also, the use of MenSCs-EVs for diagnostic purposes could enable earlier detection and more precise targeting of therapies, leading to more personalized and effective treatment strategies. MenSC-EVs represent a promising source of biomarkers for female reproductive disorders. For instance, we demonstrated that MenSC-EVs can be used as a source of biomarkers of unexplained infertility (uIF) (Vaiciuleviciute et al., 2025). These EVs were compared between healthy and uIF female groups and detected differences included alterations in cell adherence, inflammatory processes, protein metabolism of uIF patients, as compared to healthy controls, which are promising for further uIF validation in women who are not able to conceive for at least a year. Also, menstrual blood serum was characterized as a less invasive source of infertility biomarkers, where EMILIN1, TRIP6, LAMB1, LAMC1, NID1, APOB, APOA4 were detected as the main differences in uIF patients as compared to healthy controls (Brennan et al., 2025). Both MenSCs and MenSC-EVs already showed alterations in endometriosis and endometriosis-related infertility compared to healthy donors (Cordeiro et al., 2023; Zhou et al., 2020). MenSC-EVs even indicated decidual response that is critical for embryo implantation. Additionally, EVs derived from uterine fluid may serve as biomarkers for endometrial receptivity assessment in assisted reproductive technologies (Giacomini et al., 2021).

MenSC-EV-based diagnostics could offer a non-invasive alternative with significant potential for the monitoring of endometrial receptivity and pathology diagnostics of female reproductive diseases. MenSC-EVs not only share the inherent advantages of EV-based diagnostics but also offer additional benefits derived from their cellular origin. Menstrual blood collection is a non-invasive, easily accessible, and repeatable process, eliminating ethical concerns associated with other sources of reproductive tract-derived EVs. Importantly, because menstrual blood is collected during the same phase of the menstrual cycle, it minimizes variability related to hormonal fluctuations and serves as a highly localized source of biomarkers, providing a direct reflection of endometrial status (Zaheer et al., 2024).

**FIGURE 3:**
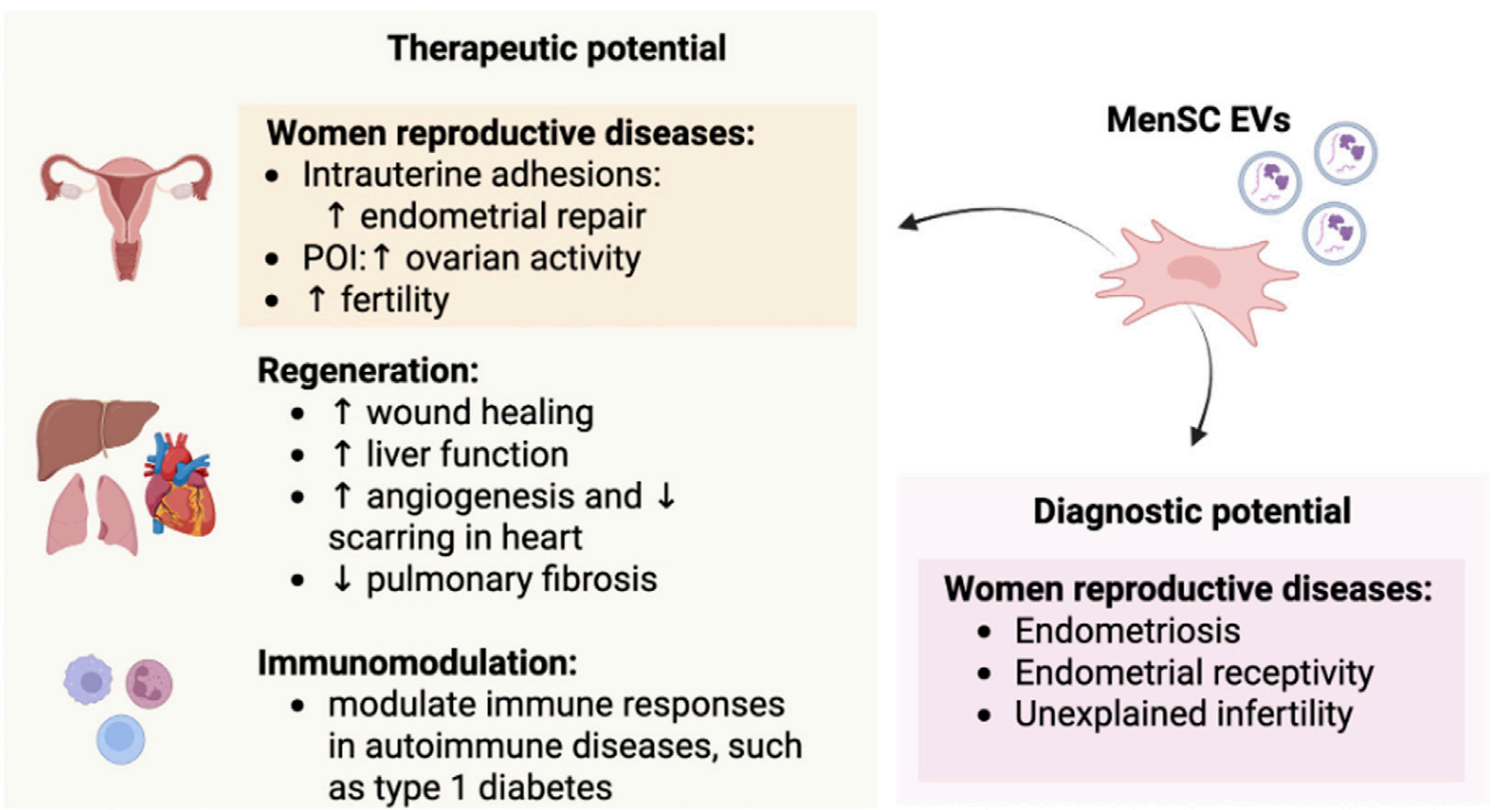
MenSC-EV therapeutic and diagnostic potential for different types of diseases and immunomodulation.

The therapeutic and diagnostic potential of MenSC- EVs is schematically visualized in Figure 3, presenting current *in vitro* and *in vivo* study discoveries.

## 6 Discussion and future directions

The uterine endometrium is a unique, fast-regenerating tissue, which plays an essential role in the female reproductive system. It has been considered as an easy-accessible source for stem cells decades ago (Borlongan et al., 2010). The endometrium undergoes over 400 cycles of regeneration during a woman’s reproductive life cycle, allowing for pregnancy, and can be continued to regenerate after menopause using hormone therapy (Tabatabaei and Ai, 2017). Endometrial stromal cells–MenSCs, have drawn attention in modern research, relating to evidence of their pluripotent-like and therapeutic properties. They offer a non-invasive alternative to traditional MSC sources and hold promise for regenerative applications, particularly through EVs, which enhance tissue repair (Chen et al., 2017; Asl et al., 2023). Even though EVs from all types of MSCs have positive effects on tissue regeneration–for instance, BMMSC-EVs showed increased muscle regeneration in a rat sarcopenia model, restored bone mass and strength in a mice osteoporosis model, regenerated cartilage, restored heart function in myocardial infarction in rat models and others (Guo et al., 2024; Wang et al., 2023; Bian et al., 2013) – MenSC-EVs show exceptional therapeutic potential in the female reproductive system, wound healing, neural, liver regeneration, and more, as discussed previously.

The potential of MenSC-EVs as a therapeutic tool for postmenopausal women is a promising field in regenerative medicine. As women age, particularly after menopause, they face a range of health challenges such as osteoporosis, muscle degeneration, skin aging, and decreased regenerative capacity across various tissues. The application of MenSCs and their EVs presents a novel approach to mitigate these age-related conditions by enhancing tissue regeneration and reducing the effects of chronic inflammation and immunosenescence, which are often observed in postmenopausal women. MenSC-EVs have shown potential in promoting tissue repair and regeneration through their cargo, which includes growth factors, cytokines, lipids, and RNAs that regulate cell survival, proliferation, and differentiation, as described above. These bioactive molecules help to modulate immune responses, stimulate tissue repair and enhance the functionality of damaged cells. Noteworthy, the ability to collect menstrual blood for autologous treatment with MenSCs is progressively reduced in elderly women, representing a limitation for their therapeutic applications. On the other hand, if these cells could be collected and cryopreserved in advance, there will always be an opportunity to use them later in the donor’s lifetime. Additionally, the concept of biobanking MenSCs, particularly from younger women, holds significant potential for future therapeutic applications. Cryopreserving MenSCs could provide a ready and accessible resource for regenerative therapies in elderly populations. Such biobanks would enable the use of autologous MenSCs and their EVs for personalized medicine in later years, overcoming the limitations associated with age-related declines in stem cell function and regenerative capacity. This approach could be particularly advantageous for postmenopausal women, as it offers the possibility of utilizing young, high-quality MenSCs for future therapies targeting conditions such as age-related diseases.

### 6.1 Limitations

Despite the promising potential of MenSC-EVs in regenerative medicine and disease diagnostics, several limitations of the current source and EVs should be acknowledged. First of all, there is a lack of long-term safety and efficacy data in all of the published studies, as most of them focus on short-term outcomes. Long-term effect of MenSC-EVs on tissue homeostasis or potential off-target response remain largely unexplored, where rigous *in vivo* studies are essential to ensure translational relevance and clinical safety. Moreover, there is a significant variability in EV isolation and characterization protocols across studies, which is an important aspect to bear in mind working with various EV sources, not only MenSC. Differences in EV isolation, filtration methods, their parameters, quantification techniques, instruments used contribute to inconsistencies in EV purity, yield and functional content. Even if the protocols are normalized, refined according to the consensus guidelines as minimal information for studies of EVs (MISEV) (Welsh et al., 2024), the variability between batch-to-batch samples is also a significant issue adapting EVs for therapeutic purposes.

Also, the use of MenSC-EVs faces regulatory, manufacturing, and bioethical challenges that need to be addressed to ensure their safe and effective use in clinical applications. On the regulatory side, the absence of specific guidelines and the complexity of proving safety, efficacy, and pharmacokinetics make clinical approvals difficult (Stawarska et al., 2024; Wang et al., 2024). Manufacturing these EVs at scale while maintaining consistency, stability, and quality remains a major obstacle (Claridge et al., 2021; Wang et al., 2024). Ethically, while menstrual blood is a non-invasive source, it has a significant ethical advantage over other stem cell sources. Issues such as informed consent and donor privacy must be carefully managed (Achmad and Götte, 2014; Savary et al., 2023). Therefore, in order to ensure reproducibility and clinical applicability, future research should prioritize standardized methodologies, explore the mechanisms underlying MenSC-EV therapeuitc actions and conduct controlled *in vivo* studies with long-term follow ups to support their safety and integration into clinical therapies.

### 6.2 Conclusion

In conclusion, MenSCs and their EVs represent a potential tool for advancing diagnostics and therapies. Their ability to promote tissue regeneration, provide diagnostic insights, and enable personalized treatments holds immense potential for improving the quality of life for women of all ages. The development of biobanks for MenSCs could further enhance the accessibility and applicability of these cells and their EVs, offering a new hope for the development of innovative treatment strategies for different conditions.
